# Parental Knowledge, Attitudes, and Perceptions Impacting Willingness to Vaccinate Against the Human Papillomavirus in Trinidad

**DOI:** 10.7759/cureus.43581

**Published:** 2023-08-16

**Authors:** Shastri Motilal, Nicholas Mohepath, Jana Moncur, Ricky Mohess, Vasthala Mohan, Shanaz Mohammed, Diana Moore, Katherina Mosca, Tisha Mulchan

**Affiliations:** 1 Department of Paraclinical Sciences, The University of the West Indies, St. Augustine, TTO

**Keywords:** cervical cancer prevention, perceived barriers, vaccine acceptance, parent survey, human papillomavirus (hpv)

## Abstract

Background

Cervical cancer remains a major cause of morbidity and mortality in young women in Trinidad and Tobago. This study aimed to determine the knowledge, attitudes, perceptions, and beliefs of Trinidadian parents toward human papillomavirus (HPV) vaccination. In addition, factors predictive of willingness to vaccinate were explored.

Methodology

In this cross-sectional study conducted between March and May 2019, a paper-based survey was self-administered to parents of children in the 5-12-year age group in seven geographically representative Trinidadian primary schools.

Results

Of the 420 questionnaires distributed, 160 were returned completed (38% response rate). General knowledge that HPV causes cervical cancer and genital warts and is spread by sexual contact was common among 81%, 71%, and 81% of parents, respectively. At least 40% of the respondents expressed uncertainty about the vaccine’s long-lasting health problems and its effectiveness in preventing genital warts and cervical cancer. Half of the parents were unsure if the vaccine was harmful. The perceptions that vaccine safety data are fabricated, drug companies cover up the dangers of vaccines, vaccine efficacy data are often fabricated, people are deceived about vaccine efficacy and safety, and conspiracy beliefs were held by 15.5%, 26.1%, 13%, 21.7%, and 28.5% of parents, respectively. There was a negative correlation between knowledge and conspiracy belief scores (ρ = -0.30, p < 0.001). Overall, 45.3% of parents were willing to immunize their children against HPV. Being informed about HPV by a health professional (odds ratio (OR) = 2.9, 95% confidence interval (CI) 1.5-5.8), knowledge of the benefits (OR = 4.6, 95% CI = 2.2-9.6), and a health professional offering the option of vaccination (OR = 3.7, 95% CI = 1.7-8.0) were associated with significantly increased odds of parents willing to vaccinate their child. The agreement that vaccine safety data are often fabricated (OR = 0.31, 95% CI = 0.12-0.84), pharmaceutical companies cover up the dangers of vaccines (OR = 0.14, 95% CI = 0.06-0.37), waiting at the clinic being time-consuming (OR = 0.37, 95% CI = 0.15-0.89), and the beliefs that adolescents are too young to get a vaccine to prevent sexually transmitted disease (OR = 0.16, 95% CI = 0.11-0.83) were associated with a significantly decreased willingness to vaccinate.

Conclusions

While general knowledge about HPV was high, there remain several areas for parental education regarding the HPV vaccine. Misbeliefs need to be addressed and multilevel interventions are needed to improve HPV vaccine uptake in our setting.

## Introduction

The human papillomavirus (HPV) is a DNA virus that is the predominant cause of cervical cancer worldwide [1]. There is a wide range of known HPV genotypes, which can be classified as low-risk and high-risk types [2]. Low-risk types (e.g., HPV 6 and 11) cause benign anogenital warts, while other types (e.g., HPV 2 and 4) are associated with common warts. High-risk types (e.g., HPV 16, 18, 31, and 33) are associated with various cancers and are implicated in the majority of cervical cancer cases [2].

Cervical cancer is the fourth most prevalent cancer globally [3]. The World Health Organization (WHO) reported 604,000 cases and 342,000 deaths in 2020 with 90% of the global mortality occurring in low and middle-income countries [3]. In Trinidad and Tobago (TT), cervical cancer was the second most common cancer in women aged 15 to 44 years and the most common cause of cancer death in this age category [4].

According to the WHO, cervical cancer is the most preventable form of cancer [3]. The mainstays of prevention are screening and vaccination [3]. These vaccines have been found to provide virtually 100% protection against cervical infections and associated oncogenic effects of HPV and, as such, have been added to the childhood immunization regimen as a proven form of primary prevention [5].

In 2013, the Ministry of Health, TT, expanded its national program on immunization by introducing the HPV vaccine as part of a school-based initiative. Sensitization workshops targeting healthcare providers, schools and parent associations, and religious groups were held [6]. However, despite the free access and availability of HPV vaccines in TT, uptake has been poor. In both males and females, HPV coverage in 2021 for the first and last doses has been estimated to be 19% and 8%, respectively [4]. There are, however, several barriers to childhood vaccination. A cross-sectional study of adolescent girls in TT found a significant knowledge gap where 63% were unaware that cervical cancer is caused by the HPV virus or that it was sexually transmitted [7]. Parental barriers toward HPV vaccines have also been explored, qualitatively illustrating themes of cervical cancer and vaccine knowledge, as well as barriers to HPV vaccine uptake [8]. As the demographic that benefits from HPV vaccination is children, surveys of parents are essential to understand the reasons for vaccine uptake or refusal. The overall goal of this study was to provide insights into the current level of parental knowledge, attitudes, beliefs, and willingness to vaccinate against HPV, and in Trinidad, and whether or not this knowledge negatively affects HPV vaccination.

## Materials and methods

Study design and setting

This was a cross-sectional study. In Trinidad, the education system is run through a network of government-assisted denominational, government-run, private institutions, and special schools which account for 59%, 24%, 15%, and 2% of all primary schools, respectively. [9] A convenient sample of seven denominational schools was selected for this study stratified by geographic location throughout Trinidad.

Sample and selection

The study population comprised parents of children aged 5-12 years attending the primary schools selected by convenience. After written permission was obtained, paper-based questionnaires were given to school principals to distribute to all parents of their pupils aged 5-12 years. A sample size of 384 was used to estimate a proportion of 50% on survey items with 95% confidence limits and a precision of 5%.

Data instrument, collection, and analysis

**Figure 1:**
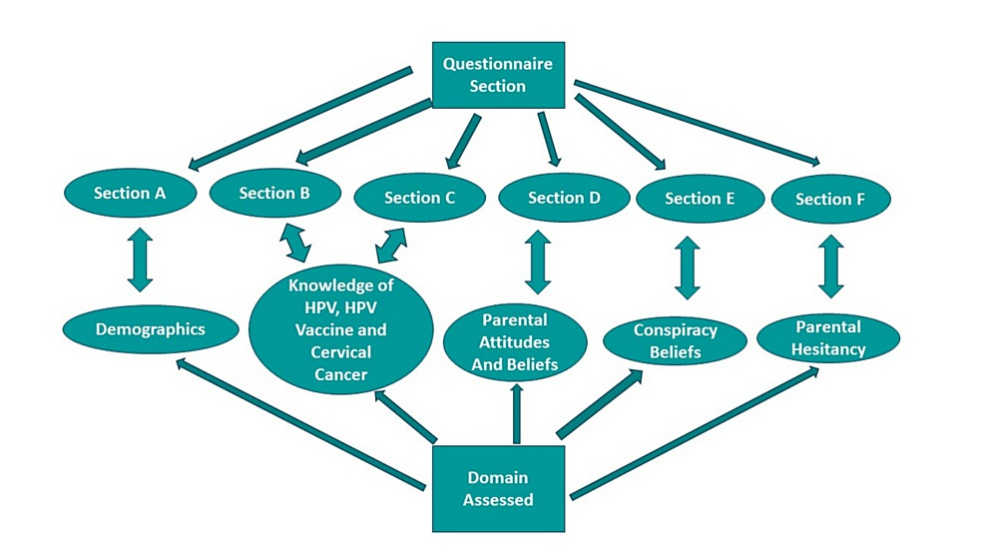
Domains of the survey tool. Questions were based on the literature for the domains of knowledge [10], attitudes and beliefs [11], vaccine conspiracy beliefs [12], and vaccine hesitancy [13].

The questionnaire was developed using four pre-existing surveys designed to ascertain views and beliefs surrounding HPV and the HPV vaccine. Figure 1 illustrates the questionnaire domains and sources used in developing each domain’s questions.

A survey conducted in Thailand that focused on parental knowledge, belief, and acceptance regarding the HPV vaccination of their daughters was used to pattern questions as it contained the majority of variables that this project sought to measure, including demographic factors such as age, gender, religious background, and education level [10]. To systematically gauge parental outlook on HPV and the HPV vaccine, the Carolina HPV Immunization Attitudes and Beliefs Scale (CHIAS) was used [11]. This tool provided several pertinent factors such as the perceived 13 potential harms of the HPV vaccine, barriers to vaccination, perceived effectiveness of the HPV vaccine, and uncertainty about the HPV vaccine. The Vaccine Hesitancy Scale and the Vaccine Conspiracy Beliefs Scales were also incorporated so that common perceptions and myths regarding vaccination could be examined [12,13]. The knowledge section with 14 questions was scored 1 point for the correct response and 0 for the incorrect response or “I don’t know,” with ideal knowledge being 14 points and poor knowledge being 0. The section on conspiracy beliefs was scored as Strongly Agree (5) to Strongly Disagree (1), with a score of 40 indicating maximal conspiracy beliefs and a score of 8 the least.

Assuming a 10% non-response rate, a total of 420 questionnaires were delivered to all schools from March to May 2019. Parents were asked to self-administer the questionnaire and return it to the school staff.

The collected data were analyzed using descriptive statistics as well as SPSS version 25.0 software (IBM Corp., Armonk, NY, USA) for statistical analysis. Descriptive data were presented using frequencies with percentages for categorical variables and medians with ranges for skewed data. Comparisons were done using Fisher’s exact and chi-squared tests for categorical and ordinal variables data. Binary logistic regression was used for exploring predictors of parents’ willingness to vaccinate their children against HPV. Spearman’s correlation was used for the association between non-normal continuous variables. A P-value <0.05 was used to indicate statistical significance.

Ethics

This study was granted approval by the Ethics Committee of the University of the West Indies, St. Augustine Campus (approval number: CEC869/02/19). All participating parents signed informed consent.

## Results

Demographics

Of the 420 questionnaires distributed, 161 were returned completed (38% response rate). The majority of parents were mothers (78%) of female students (58%). The age categories of the parents were 25-30 years (8%), 31-35 years (20%), and >35 years (72%) The age categories of the respondents’ children were 5-8 years (51%) and 9-12 (49%) years. The religions of Hinduism (40%), Christianity (32%), and Islam (23%) were most represented in the study sample. Two-thirds of parents attained tertiary level education, with 71% employed full-time.

Knowledge about HPV and cervical cancer

Table 1 displays parental knowledge regarding HPV and cervical cancer.

**Table 1:** Parental responses on knowledge about HPV and cervical cancer. *: Correct answer. HPV: human papillomavirus

Knowledge-based questions	True	False	I don’t know
HPV infection is contracted by sexual contact	^*^131 (81.4%)	11 (6.8%)	-
People can transmit HPV to their partner(s) even if they have no symptoms of infection	^*^129 (80.1%)	6 (3.7%)	26 (16.1%)
Having multiple sexual partners increases the risk of HPV infection	^*^132 (82%)	10 (6.2%)	19 (11.8%)
Sexual intercourse at an early age increases the risk of HPV infection	^*^103 (64%)	15 (9.3%)	43 (26.7%)
Genital warts are caused by HPV infection	^*^114 (70.8%)	12 (7.5%)	35 (21.7%)
Most people with genital HPV have no visible signs or symptoms	^*^101 (62.7%)	23 (14.3%	37 (23%)
Vaginal douching after intercourse can prevent HPV infection	10 (6.2%)	^*^101 (62.7%)	50 (31.1%)
HPV infection can be treated with antibiotics	47 (29.2%)	^*^59 (36.6%)	55 (34.2%)
Smoking increases the risk of cervical cancer	^*^80 (49.7%)	28 (17.4%)	53 (16%)
HPV infection can cause cervical cancer	^*^130 (80.7%)	6 (3.7%)	25 (15.5%)
Cervical cancer can cause bleeding after sexual intercourse	^*^95 (59%)	13 (8.1%)	53 (32.9%)
A pap smear only needs to be done in women with vaginal discharge or bleeding	11 (6.8%)	^*^139 (86.3%)	11 (6.8%)
Unmarried women are not supposed to have a pap smear	9 (5.6%)	^*^140 (87%)	12 (7.5%)
Cervical cancer symptoms commonly include vaginal discharge or bleeding even in the early stages	93 (57.8%)	^*^17 (10.6%)	51 (31.7%)

The median knowledge score was 10, ranging from 0 to 14. Most parents (93.2%) had heard about HPV previously, with common sources being friends, advertisements, the internet, medical office, and brochures at 34.6%, 21.8%, 19.2%, 14.7%, and 8.3%, respectively. Two-thirds (65%) reported knowing about the benefits of the HPV vaccine and a minority (24%) stated that a health professional provided the option of their child receiving the vaccine.

Of the parents who knew about HPV, 69% attained tertiary-level education compared to 36% who did not hear about HPV (p = 0.04). There were no other significant associations between knowledge of HPV and any other demographic variable (all p-values >0.05).

Most respondents selected the correct options. However, the majority agreed that vaginal discharge or bleeding even in the early stages of cervical cancer is a common presentation which is false. Approximately half (49.7%) of the sample agreed that smoking increases the risk and 36.6% agreed that antibiotics can be used to treat HPV infection. Agreement with the latter was significantly associated (p = 0.013) with being a mother (91.3%) compared to disagreement with this statement (67.2%).

Beliefs and attitudes regarding HPV and HPV vaccine

Table 2 highlights the beliefs and attitudes regarding HPV and its vaccine.

**Table 2:** Parental attitudes and beliefs responses about HPV and the HPV vaccine. *: Modal response. HPV: human papillomavirus

Beliefs and attitudes about HPV and vaccine	Strongly agree	Agree	Unsure	Disagree	Strongly disagree
The HPV vaccine might cause lasting health problems	11 (6.8%)	25 (15.5%)	*74 (46.0%)	42 (26.1%)	9 (5.6%)
If children get the HPV vaccine, they may be more likely to have sex when they are teenagers	2 (1.2%)	15 (9.3%)	26 (16.1%)	*76 (47.2%)	42 (26.1%)
There is a risk for young men and women to contract HPV	45 (28.0%)	*83 (51.6%)	28 (17.4%)	4 (2.5%	1 (0.6%)
There is a risk for young women to contract cervical cancer	39 (24.2%)	*81 (50.3%)	29 (18.0%)	9 (5.6%)	3 (1.9%)
HPV infection is a serious health concern	68 (42.2%)	*75 (46.6%)	15 (9.3%)	3 (1.9%)	-
Cervical cancer is a serious disease	*105 (65.2%)	50 (31.1%)	5 (3.1%)	1 (0.6%)	-
The HPV vaccine is effective in preventing genital warts	26 (16.1%)	56 (34.8%)	*69 (42.9%)	9 (5.6%)	1 (0.6%)
The HPV vaccine is effective in preventing cervical cancer	26 (16.1%)	*69 (42.9%)	53 (32.9%)	13 (8.1%)	-
The efficiency and effectiveness of the HPV vaccine is unclear	15 (9.3%)	34 (21.1%)	*67 (41.6%)	43 (26.7%)	2 (1.2%)
The HPV vaccine is harmful	4 (2.5%)	9 (5.6%)	*82 (50.9%)	57 (35.4%)	9(5.6%)
Women who have been HPV vaccinated should have a pap smear done annually	30 (18.6%)	*87 (54.0%)	39 (24.2%)	5 (3.1%)	-
HPV vaccination decreases condom use	2 (1.2%)	9 (5.6%)	31 (19.3%)	*71 (44.1%)	48 (29.8%)
HPV vaccination increases awareness of sexually transmitted diseases	27 (16.8%)	*91 (56.5%)	25 (15.5%)	15 (9.3%)	3 (1.9%)
Getting vaccines is a good way to protect my child from disease	46 (28.6%)	*81 (50.3%)	26 (16.1%)	8 (5.0%)	-
Generally, I do what my doctor or healthcare provider recommends about vaccines for my children	49 (30.4%)	*93 (57.8%)	8 (5.0%)	9 (5.6%)	2 (1.2%)
My child does not need vaccines for diseases that are not common	7 (4.3%)	18 (11.2%)	26 (16.1%)	*79 (49.1%)	31 (19.3%)

Approximately one-fifth (22.3%) of the study sample felt that the HPV vaccine might cause health problems. Overall, 59% felt the HPV vaccine is effective in preventing cervical cancer, and 78.3% felt vaccination is a good way to prevent disease. Over 70% disagreed that the HPV vaccine would make children more likely to have sex as teenagers while 10.5% believed it would. Tertiary education was significantly associated with the agreement that the HPV vaccine is effective in preventing cervical cancer (odds ratio (OR) = 12.3, 95% confidence interval (CI) = 7.0-21.6). 

Conspiracy beliefs regarding HPV Vaccine

Table 3 shows the conspiracy beliefs held by parents in this survey.

**Table 3:** Parental conspiracy beliefs responses. *: Modal response. HPV: human papillomavirus

Parental conspiracy beliefs scale	Strongly agree	Agree	Unsure	Disagree	Strongly disagree
Vaccine safety data is often fabricated	5 (3.1%)	20 (12.4%)	*83 (51.6%)	50 (31.1%)	3 (1.9%)
Immunization of children is harmful and this fact is covered up	-	13 (8.1%)	40 (24.8%)	*84 (52.2%)	24 (14.9%)
Pharmaceutical companies cover up the dangers of vaccines	9 (5.6%)	33 (20.5%)	*66 (41.0%)	44 (27.3%)	9 (5.6%)
People are deceived about vaccine efficacy	7 (4.3%)	28 (17.4%)	*66 (41.0%)	50 (31.1%)	9 (6.2%)
Vaccine efficacy data is often fabricated	10 (6.2%)	11 (6.8%)	*84 (52.2%)	50 (31.1%)	6 (3.7%)
People are deceived about vaccine safety	6 (3.7%)	40 (24.8%)	*54 (33.5%)	*54 (33.5%)	7 (4.3%)
The government is trying to cover up the link between vaccines and autism	7 (4.3%)	6 (3.7%)	*78 (48.4%)	61 (37.9%)	9 (5.6%)
The HPV vaccine is being pushed to make money for drug companies	4 (2.5%)	13 (8.1%)	*72 (44.7%)	65 (40.4%)	7 (4.3%)

The median conspiracy belief score was 22 ranging from 8 to 38. There was a negative correlation between knowledge and conspiracy belief scores (ρ = -0.30, p < 0.001) The perceptions that vaccine safety and efficacy data are fabricated, drug companies cover up dangers of vaccines, and people are deceived about vaccine efficacy and safety were held by 15.5%, 26.1%, 13%, 21.7%, and 28.5% of parents, respectively. Regarding a possible link between vaccines and autism, 8% believed that this was being covered up. Similarly, 8.1% also agreed that immunization of children, in general, is harmful and is being covered up.

Parental hesitancy about HPV and its vaccine

Parental hesitancy was assessed in this study, as shown in Table 4.

**Table 4:** Parental hesitancy responses regarding HPV and the HPV vaccine. *: Modal response. HPV: human papillomavirus

Parental hesitancy factors about HPV and the HPV vaccine	Strongly agree	Agree	Unsure	Disagree	Strongly disagree
Adolescents are too young to get a vaccine for sexually transmitted infections like HPV	12 (7.5%)	10 (6.2%)	29 (18.0%)	*78 (48.4%)	32 (19.9%)
The HPV vaccine is so new that I want to wait a while before deciding if my child should be vaccinated	9 (5.6%)	*60 (37.3%)	33 (20.5%)	47 (29.2%)	12 (7.5%)
I don’t have enough information about the HPV vaccine to decide if my child should be vaccinated	30 (18.6%)	*66 (41.0%)	16 (9.9%)	40 (24.8%)	9 (5.6%)
Other parents in my community are getting their children vaccinated	3 (1.9%)	23 (14.3%)	*113 (70.2%)	15 (9.3%)	7 (4.3%)
It is difficult to find a health center at which the HPV vaccine is available	4 (2.5%)	15 (9.3%)	*84 (52.2%)	48 (29.8%)	10 (6.2%)
Waiting at a health center for vaccination is time-consuming	49 (30.4%)	*63 (39.1%)	21 (13.0%)	22 (13.7%)	6 (3.7%)
The HPV vaccine might cause short-term problems, like fever or discomfort	8 (5.0%)	41 (25.5%)	*95 (59.0%)	13 (8.1%)	40 (25%)
Every child should be given the HPV vaccine	15 (9.3%)	47 (29.2%)	*72 (44.7%)	22 (13.7%)	5 (3.1%)
I have considered vaccinating myself against HPV	18 (11.2%)	*56 (34.8%)	46 (28.6%)	35 (21.7%)	6 (3.7%)
I have considered having my child vaccinated against HPV	16 (9.9%)	*57 (35.4%)	52 (32.3%)	29 (18.0%)	7 (4.3%)
If the government implemented HPV vaccination for every child in Trinidad and Tobago I would support it	29 (18.0%)	59 (36.6%)	*62 (38.5%)	6 (3.7%)	5 (3.1%)
I think the HPV vaccine is unsafe	2 (1.2%)	4 (2.5%)	*84 (52.2%)	57 (35.4%)	14 (8.7%)
The HPV vaccine can cause adverse effects	4 (2.5%)	17 (10.6%)	*99 (61.5%)	33 (20.5%)	8 (5.0%)
It is problematic that the HPV vaccine requires three injections	11 (6.8%)	23 (14.3%)	*74 (46.0%)	48 (29.8%)	5 (3.1%)
New vaccines carry more risks than older vaccines	2 (1.2%)	32 (19.9%)	*93 (57.8%)	28 (17.4%)	6 (3.7%)
I am concerned about the adverse effects of vaccines	26 (16.1%)	*72 (44.7%)	34 (21.1%)	25 (15.5%)	4 (2.5%)

The majority of respondents agreed that waiting at a health center for vaccination is time-consuming (69.5%), they are concerned about the adverse effects of vaccines in general (60.8%), and they don’t have enough information about the HPV vaccine to decide whether to vaccinate their child (59.6%). Interestingly, if the government implemented HPV vaccination for every child in TT, 54.6% of parents would support it.

There was more uncertainty regarding certain beliefs as more parents were unsure if other parents are vaccinating their children (70.2%), the HPV vaccine causing adverse effects (61.5%), and the HPV vaccine carrying short-term problems, such as fever or discomfort (59%). The perception that newer vaccines carry more risk (57.8%) and difficulty finding a health center where the vaccine is available (52.2%) were also noted.

Predictors of willingness to vaccinate against HPV

Overall, 45.3% of the sample had considered having their child vaccinated against HPV. There were no significant associations between demographics and willingness to vaccinate against HPV. Table 5 depicts perceptual factors and their associations with the willingness to vaccinate against HPV.

**Table 5:** Perceptual barriers to willingness to vaccinate against HPV. *: Referent category. HPV: human papillomavirus

Barriers to vaccination against HPV	Unadjusted odds ratio for vaccinating the child against HPV (95% confidence limits)	P-value
Vaccine safety data is often fabricated.		
Strongly agree/Agree	0.31 (0.12–0.84)	0.021
Unsure	0.16 (0.08–0.34)	<0.001
Disagree/Strongly disagree*	1	
Pharmaceutical companies cover up the dangers of vaccines		
Strongly agree/Agree	0.14 (0.06–0.37)	<0.001
Unsure	0.57 (0.27–1.19)	0.135
Disagree/Strongly disagree*	1	
People are deceived about vaccine efficacy. Strongly agree/Agree	0.16 (0.06–0.41)	<0.001
Unsure	0.25 (0.12–0.52)	<0.001
Disagree/Strongly disagree*	1	
Adolescents are too young to get a vaccine for sexually transmitted infections like HPV		
Strongly agree/ Agree	0.30 (0.11–0.83)	0.020
Unsure	0.21 (0.08–0.55)	0.002
Disagree/Strongly disagree*	1	
The HPV vaccine is so new that I want to wait a while before deciding if my child should be vaccinated		
Strongly agree/Agree	0.20 (0.10–0.43)	<0.001
Unsure	0.13 (0.05–0.34)	<0.001
Disagree/Strongly Disagree*	1	
It is difficult to find a health center at which the HPV vaccine is available		
Strongly agree/Agree	0.47 (0.17–1.36)	0.163
Unsure	0.24 (0.12–0.48)	<0.001
Disagree/Strongly disagree*	1	
Waiting at a health center for vaccination is time consuming		
Strongly agree/Agree	0.37 (0.15–0.89)	0.026
Unsure	0.15 (0.04–0.53)	0.003
Disagree/Strongly disagree*	1	
It is problematic that the HPV vaccine requires three injections		
Strongly agree/Agree	0.21 (0.08–0.53)	0.001
Unsure	0.07 (0.03–0.17)	<0.001
Disagree/Strongly disagree*	1	

Perceived fabrication of data from pharmaceutical companies on safety and effectiveness was negatively correlated with the willingness to vaccinate. Other factors included adolescent age as being too young, HPV vaccine novelty, waiting times at health centers, and accessibility of the vaccine. Unwilling parents were also more likely to agree that it is problematic that the HPV vaccine requires three injections. Those who agreed that adolescents are too young to get a vaccine for a sexually transmitted disease were also less likely to vaccinate their children than those who disagreed (p < 0.05 for all).

Conversely, there were several factors that were associated with an increased willingness to vaccinate against HPV. Being informed about HPV by a health professional, the knowledge of the benefits, and a health professional offering the option of vaccination were associated with significantly increased odds of parents being willing to vaccinate their child (p < 0.05 for all). In additional analyses, the knowledge score was positively correlated (OR = 1.20, 95% CI = 1.08-1.34) while the conspiracy belief score was negatively associated (OR = 0.86, 95% CI = 0.79-0.92) with the willingness to vaccinate against HPV.

## Discussion

This study was done in Trinidad to examine parental knowledge, attitudes, beliefs, and willingness to vaccinate against HPV. Another local study highlighted limited awareness of Trinidadian women regarding HPV and its association with cancer [14]. However, our study further examined information about HPV and its vaccine, parental acceptance and misconceptions, and related these factors to the willingness to vaccinate. Most respondents had knowledge of HPV as the cause of cervical cancer, the method of spread, and the seriousness of the disease. A similar study conducted in Manchester, United Kingdom, examined parental consent and potential vaccination in eight secondary schools and showed strong connections between vaccination and ethnicity and religion of parents, as well as parental views that the vaccine is both safe and effective [15]. Our study established a separate demographic relationship, where it showed a positive association between knowledge of HPV and tertiary-level education.

This study found a lack in select areas of knowledge, particularly regarding the use of antibiotics as HPV treatment and that early-stage cervical cancer is asymptomatic. The lack of clinical information suggests that parents have limited detailed knowledge about signs, symptoms, treatment, and preventative measures. This mirrors the findings of a study that assessed the knowledge and attitudes of people of African descent from two countries, the United States and the Bahamas. This study showed that the majority of respondents from both countries had heard of HPV (United States: 89.5% vs Bahamas: 61.5%) and knew of its relationship with cervical cancer, but showed a lack of knowledge regarding genital HPV infections being asymptomatic (21-36%) and HPV causing genital warts (28-33%) [16]. Our survey showed uncertainty about HPV vaccination causing lasting health problems, its effectiveness in preventing genital warts, its overall efficiency and effectiveness, and its harms. These areas represent opportunities for education.

Significant contributors to vaccination hesitancy found in our study were access and availability. Three studies done in the Lao People’s Democratic Republic regarding factors affecting diphtheria, measles, and routine vaccination supported low accessibility and availability as the main factors in non-vaccination, even though vaccination is free [17-19]. A study conducted in the United Kingdom that examined factors to improve vaccine uptake found that new methods of offering immunization and utilization of venues that are accessible to parents, such as shopping malls, can reduce logistical barriers to vaccination [20]. This may be applicable to the local setting where HPV vaccination is available free as a school-based program. The HPV vaccine is also available in Trinidad at a cost in the private sector; however, the high price may be a deterrent to those who wish to obtain the vaccine outside the public system. Decreased financial barriers, incentives, and interventions targeting both providers and patients may also be another mechanism to improve HPV vaccine uptake [21].

In this study, conspiracy beliefs were negatively correlated with willingness to vaccinate against HPV, findings consistent with other studies in various settings [22-25]. Knowledge was negatively associated with conspiracy beliefs in this survey. The internet and social media can be a source of objective verifiable facts or misinformation. The portrayal of mixed HPV vaccine information on popular social media platforms has been described [26-28]. Exposure of adolescents and young adults who are popular users of these platforms to misinformation can dissuade them from taking the HPV vaccine. The need for public health authorities to monitor online platforms for anti-vaccine rhetoric and create mitigating campaigns has been highlighted [29].

Conversely leveraging the power of social media may also have a positive impact on HPV vaccine uptake. This recommendation is supported by a study, which showed some positive correlation between social media and HPV vaccination [30]. Other digital interventions have also been shown to be cost-effective means to educate parents and adolescents in an engaging manner [31]. Additionally, culturally targeted interventions may improve the poor HPV vaccine uptake seen locally [32]. Given Trinidad’s influence of its indigenous music on health education, role models and artists may also play a role in promoting vaccine education and debunking myths [33].

Our study found that parents who were informed about HPV vaccination by health professionals and those who knew about the role of HPV in causing cancer showed greater willingness to vaccinate. This places importance on the role of healthcare professionals in increasing vaccine uptake by providing information on vaccine benefits and risks associated with non-vaccination. A systematic review highlighted several techniques that healthcare professionals can use to stimulate HPV vaccine uptake [34]. Another review, of strategies to increase HPV vaccination rates in primary care settings, highlighted the role of multiple interventions at varying points in time surrounding the visit [35]. A similar promotion of the HPV vaccine in Trinidad’s primary care system, where the national immunization program is mostly delivered, can also increase HPV vaccine uptake. Targeting Trinidad’s low HPV vaccine uptake through several means may be best as a trial showed that multilevel interventions for the clinic, provider, and parent increased HPV vaccine uptake [36].

Strengths and limitations

This study adds to the Trinidadian literature on parental views of HPV and the HPV vaccine. Several validated scales were used to develop the questionnaire which was used to assess parental knowledge, attitudes, perceptions, barriers, and conspiracy beliefs. It provided useful insights into possible barriers which can guide further similar studies, as well as possible interventions to increase HPV vaccine uptake.

Non-response bias was the main limitation of this study, as the self-administration of questionnaires resulted in a low response rate. Selective schools were unable to complete the quota of questionnaires received due to the subsequent release of results for the Secondary Entrance Assessment. This is an examination taken by students, 11-12 years old, across TT, for admission to secondary school. A second limitation was the non-generalizability of the findings to all parents in Trinidad. Surveying selected denominational schools may not be representative of the views of parents of government-based and private school children. Third, the study’s findings were reported four years after the initial survey. While this may suggest outdated data, a second dose uptake rate of less than 10%, eight years after the implementation of a freely accessible HPV vaccine, suggests HPV vaccine hesitancy is a long-standing problem. Lastly, selection bias through convenience sampling and social desirability bias could have occurred in parental responses.

## Conclusions

Despite good parental knowledge, several parental beliefs and factors that impacted the willingness to vaccinate against HPV in Trinidad were found. This study highlights the need for targeted education to address gaps in knowledge, particularly regarding HPV treatment and early-stage cervical cancer symptoms. Factors such as limited access and availability were identified as significant contributors to vaccination hesitancy, suggesting the importance of implementing interventions to improve vaccine uptake, such as utilizing more accessible venues and reducing financial barriers.

This study also emphasizes the negative impact of conspiracy beliefs on willingness to vaccinate, calling for monitoring, and countering misinformation on online platforms. Leveraging the influence of social media and cultural interventions may offer promising avenues to promote vaccine education and combat myths. Health professionals play a crucial role in increasing vaccine acceptance by providing comprehensive information on vaccine benefits and risks. Despite some limitations, this research underscores the persistent issue of HPV vaccine hesitancy in Trinidad and highlights the necessity for ongoing, multilevel efforts to increase HPV vaccination rates.
